# Cases of Atypical Lymphangiomas in Children

**DOI:** 10.1155/2014/626198

**Published:** 2014-09-29

**Authors:** Prashant K. Minocha, Lakhan Roop, Rambachan Persad

**Affiliations:** Department of Paediatric Surgery, San Fernando General Hospital, Trinidad, Trinidad and Tobago

## Abstract

*Background*. Lymphatic malformations or lymphangiomas are rare benign hamartomas that result from maldevelopment of primitive lymphatic sacs. They are most frequently found in the neck and axilla, while intra-abdominal and mediastinal lymphangiomas are uncommon. These are primarily tumours of infancy and childhood and are successfully treated with surgical excision. *Summary of Cases*. Five cases of lymphangioma comprising three intra-abdominal lymphangiomas and two unilateral axillary lymphangiomas presenting at one institution in Trinidad W.I. between 2005 and 2012 were examined. The presentations, location, workup, treatment, and outcome of these patients were studied. *Conclusion*. This paper discusses a range of extracervical lymphangioma cases seen at San Fernando General Hospital, Trinidad W.I. We report three intra-abdominal cases and the most common clinical presentations were abdominal pain and distension. Also two axillary cases were reported, which presented as painless axillary masses. The major concerns for excision of axillary lymphangioma by parents and surgeons were cosmesis and feasibility of complete resection without disruption of developing breast tissue and axillary vessels. We believe that ultrasound scan is very good at detection of the lesion, while CT is better at determining tumour content and planning for the operation. It is our opinion that complete surgical excision can be achieved.

## 1. Introduction

Lymphangiomas are benign lesions characterized by proliferation of lymphatic vessels. Approximately 50% are present at birth and 90% are diagnosed before the age of 2 [1]. They are most frequently found in the neck (75%) and axilla (15%) [2], while only 10% are found in the mediastinum and abdominal cavity [3, 4] including mesentery, retroperitoneal areas, and bones [5]. Retroperitoneal lymphangiomas are extremely rare comprising 1% of all lymphangiomas [6]. Lymphangiomas are successfully treated with surgical excision; however, there have been cases of recurrence with patients who have undergone incomplete excision (Table 1).

## 2. Case Descriptions

### 2.1. Case 1

The first patient was a 6-year-old female who at 6 weeks of life had a surgically resected cystic lymphangioma of the sigmoid colon (with primary colorectal anastomosis and appendectomy). She was previously well; however, she began experiencing symptoms of intermittent periumbilical abdominal pain, accompanied by vomiting, constipation, and abdominal distension, which necessitated two separate admissions. On the first admission, she was treated for acute intestinal obstruction, which spontaneously resolved after several days at the hospital. Three months later, she presented with periumbilical pain for two days followed by vomiting, abdominal distension, and headaches. On examination, her abdomen was noted to be mildly distended, but no discrete masses were palpated. She had no family history of malignancy or congenital malformations. On this admission, her haemoglobin (Hb) was found to be 5 gm/dL and she required transfusion of 250 mLs of packed red cells. Her posttransfusion Hb increased to 9.5 gm/dL. Abdominal X-ray did not show any bowel dilation or air fluid levels. Given the patient's past surgical history, there was a high index of suspicion for recurrence of the intra-abdominal lymphangioma. Other differentials included intestinal obstruction and mesenteric adenitis. A computed tomography (CT) scan of the abdomen with IV (intravenous) contrast was performed which showed a cystic mass on the colon and she had surgical intervention. Findings of the surgery included a retroperitoneal cystic lymphangioma enclosing a 50 mL organized clot in the left iliac fossa, multiple peritoneal adhesions, mesenteric windows, adhesions enclosing small bowel into artificial sac, and a ventral incisional hernia. Histology confirmed cystic lymphangioma. The patient has been followed up with regular ultrasound scans (USS) for the past 9 years and there has been no evidence of recurrence or intestinal obstruction thus far.

### 2.2. Case 2

**Figure 1:**
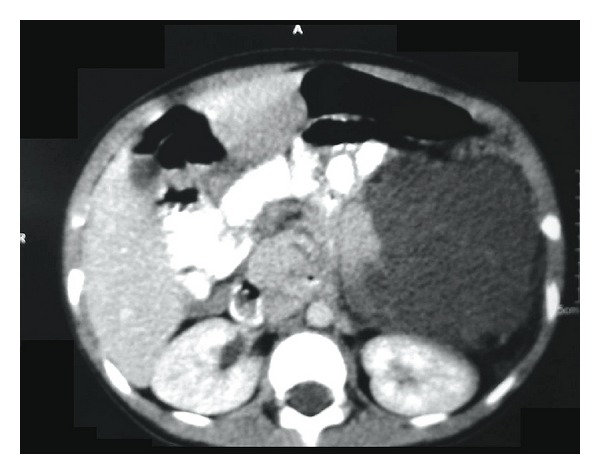
CT of the abdomen showing a 30 cm × 10 cm left sided isodense retroperitoneal mass.

A 4-year-old male presented with 2-day history of periumbilical pain and a past history of occasional constipation without any significant past medical history and no family history of malignancy or congenital abnormalities. On examination he was found to have a mildly distended, nontender abdomen with diffuse left flank firmness, but no discrete mass was appreciated. The patient continued to have colicky abdominal pain but no constipation or vomiting. CT scan abdomen with IV contrast was performed which showed a 30 cm by 10 cm left sided isodense retroperitoneal mass attached to the lower pole of left kidney (Figure 1). Based on imaging, the possible differential diagnoses included neuroblastoma and cystic lymphangioma. The patient underwent an exploratory laparotomy which revealed a 20 cm by 30 cm multiloculated cystic mass arising retroperitoneally from the coeliac plexus of lymph. The hilum of the cyst was anterior to splenic artery displacing pancreas laterally to the right. The histology of the specimen confirmed intra-abdominal cystic lymphangioma. Yearly follow-up with USS for the past 8 years has shown no recurrence thus far.

### 2.3. Case 3

**Figure 2:**
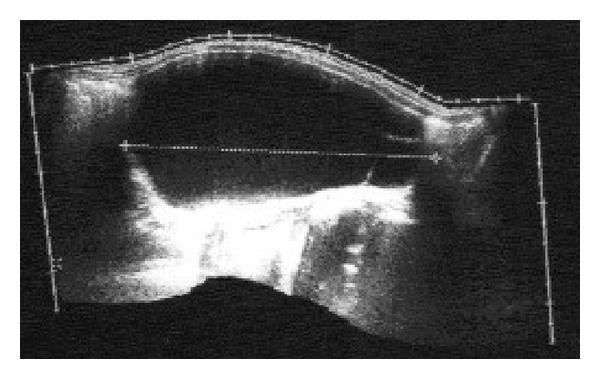
Abdominal ultrasound showing a 14 cm by 7 cm by 12 cm fluid filled structure in the left half of the abdomen.

**Figure 3:**
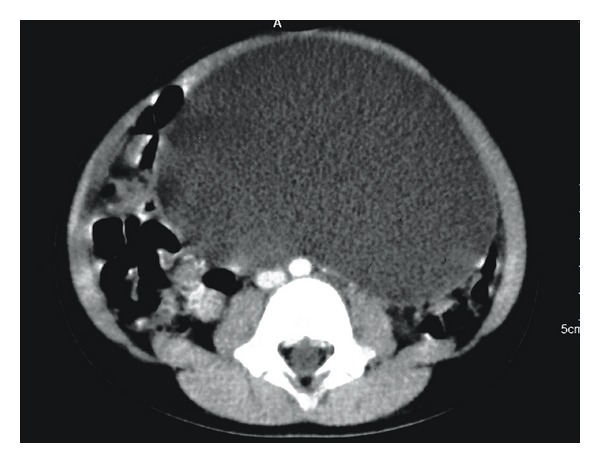
Abdominal CT showing a 13 cm × 8 cm × 12 cm cystic abdominal mass.

**Figure 4:**
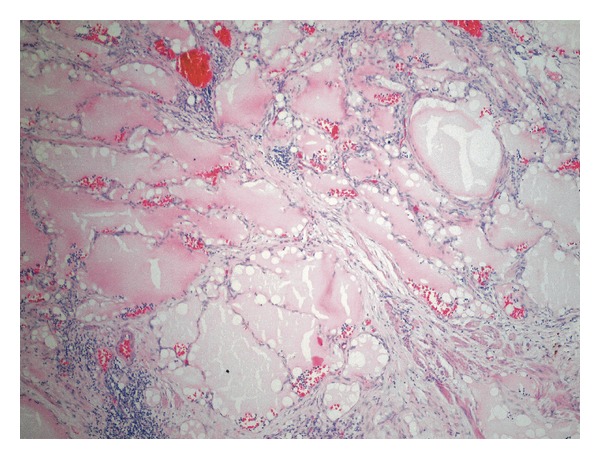
Histology showing a mixture of lymph vessels and smooth muscle, with lymphatic channels containing blood and lymphoid cells (magnification ×100).

The patient was a 4-year-old female who was referred from a rural health center with 2-week history of progressive abdominal distension. She was otherwise asymptomatic with a positive history of passing stool and flatus. There was no significant past medical history or history of prior abdominal surgery or trauma. She had no family history of malignancy or congenital abnormalities. On abdominal examination, a mass arising from the pelvis was palpable. An abdominal USS revealed a 14 cm by 7 cm by 12 cm fluid filled structure in the left half of the abdomen extending into the pelvic cavity (Figure 2). Abdominal CT with IV contrast showed a 13 cm by 8 cm by 12 cm cystic abdominal mass that appeared to arise from the pelvis extending into the abdomen and displacing the bowel bilaterally (Figure 3). Two days subsequent to admission, the patient had exploratory laparotomy and cystectomy. A 15 cm by 12 cm mesenteric cyst containing chocolate coloured fluid was found at the splenic hilum surrounded by a jejunal loop and bordered by the tail of the pancreas. She had uneventful recovery with no evidence of recurrence to date. Histology showed a mixture of lymph vessels and smooth muscle, features suggestive of lymphangioma (Figure 4).

### 2.4. Case 4

**Figure 5:**
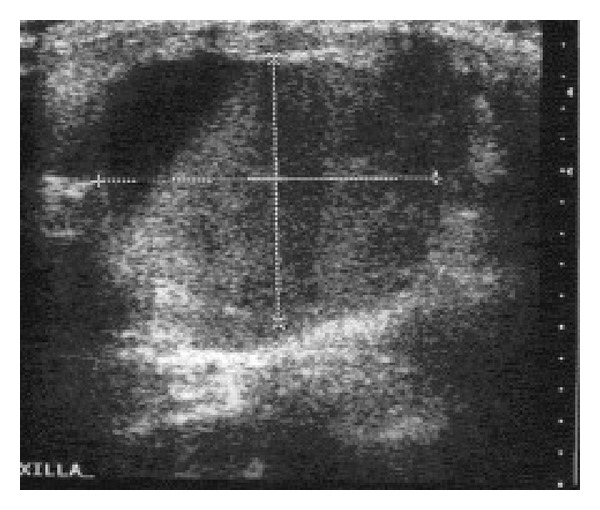
Ultrasound of the chest showing a heterogeneous solid cystic mass in the left axilla.

**Figure 6:**
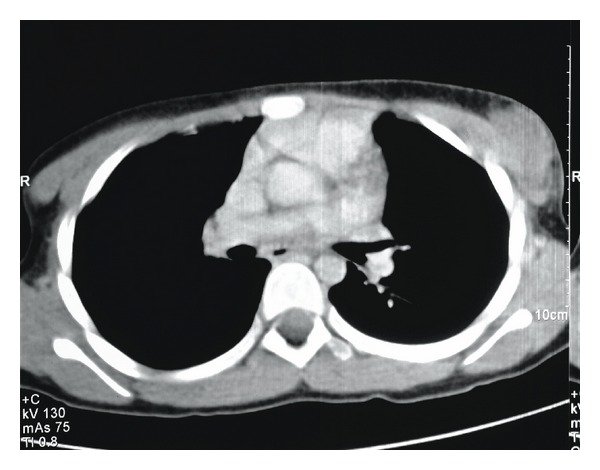
CT of the chest showing several soft tissue lesions noted laterally and anterior to the left pectoralis major measuring 4 cm × 3 cm × 1 cm.

The patient was an 8-year-old female who presented with a 4-day history of a swelling to the left side of her chest that was increasing in size and she was otherwise asymptomatic. She had no medical problems and no significant family history. Interestingly, she was known to have a lymph node in the same area 7 years prior and was scheduled for surgical excision, but it resolved and hence no surgery was done at that time. On examination, she was found to have a 3 cm by 3 cm firm, smooth mass in her left axilla with no surrounding lymphadenopathy. USS of the chest showed a heterogeneous solid and cystic mass in the left axilla (Figure 5). The patient had needle aspiration which revealed a bloody aspirate and this was followed by a course of oral antibiotics; however, the swelling failed to resolve. CT scan of the chest with contrast done subsequently demonstrated a left axillary cystic mass with no intrathoracic extension (Figure 6). She had complete surgical excision of the cystic mass. However, the proximity of the lesion to developing breast tissue posed a challenge to surgical resection. Follow-up in clinic for the past 3 years has shown symmetrical development of breasts with no deformity to the left breast. Histology confirmed the diagnosis of lymphangioma.

### 2.5. Case 5

**Figure 7:**
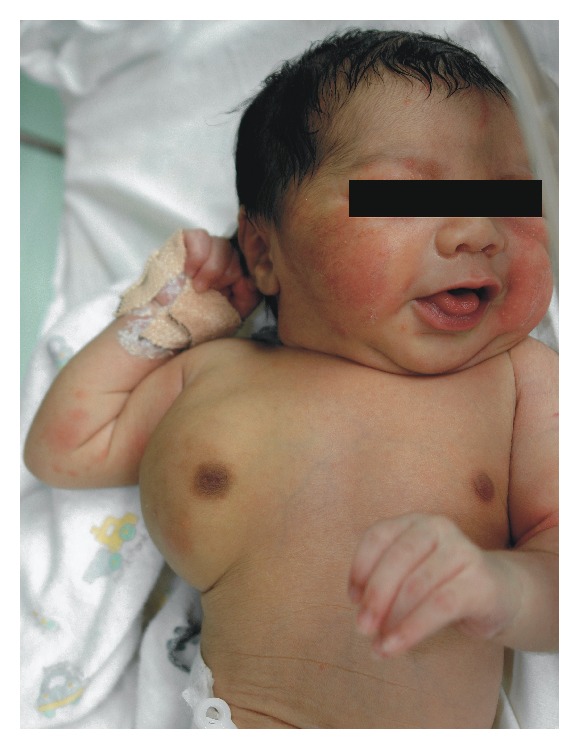
Picture showing right chest swelling shortly after birth.

**Figure 8:**
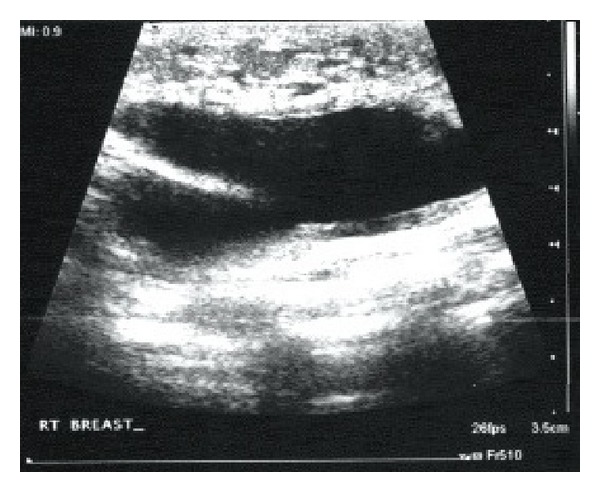
Ultrasound of the chest/axilla showing a cystic mass with septations in the right lateral chest wall near the right axilla, 5 cm × 4 cm.

**Figure 9:**
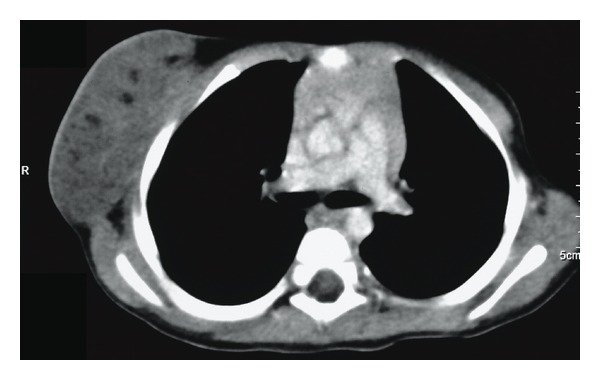
CT of the chest showing an 8 cm × 7 cm × 3 cm enhancing mixed density mass in the right chest wall.

This male patient was referred to us at birth with a right chest swelling (Figure 7). USS of the chest revealed a predominantly cystic mass with septations in the right lateral chest wall near the right axilla, 5 cm by 4 cm (Figure 8). Differential diagnoses included axillary lymphangioma and unilateral gynaecomastia and he was scheduled for regular follow-up at our outpatient clinic. The patient had no medical problems and no significant family history. Monthly follow-up revealed that the mass was progressively decreasing in size. However at one-year follow-up, mother noticed an increase in size with the mass measuring 6 cm by 5 cm on physical examination. The patient was scheduled for elective surgical excision pending CT scan. Chest CT with contrast revealed 8 cm by 7 cm by 3 cm enhancing mixed density mass in the right chest wall abutting the pectoralis major muscle (Figure 9). Surgical excision performed at 14 months showed 7 cm by 7 cm cystic mass in the right axilla. The surgical site healed well and follow-up with USS for the past 2 years has been uneventful thus far. Histology confirmed the diagnosis of lymphangioma.

## 3. Discussion

Lymphatic malformations or lymphangiomas are congenital anomalies composed of dilated lymphatic channels. They are filled with a proteinaceous fluid and generally do not have connections to the normal lymphatic system [7]. Lesions can be macrocystic (>1 cm), microcystic (<1 cm), or mixed [8, 9]. Lymphangiomas can be divided into lymphangioma circumscriptum, which are superficial, cutaneous lesions, and cavernous lymphangioma, which are more deep-seated.

Cavernous lymphangiomas occur in areas of loose connective tissue, areola, and are found primarily during infancy, and most are diagnosed by the age of 2 [1]. They are commonly located in the head, neck, and axilla and are rarely intra-abdominal. It is believed that lymphangiomas arise due to failure of connection between primitive lymphatic sacs and the surrounding lymphatic channels due to abnormalities in embryonic development [10]. This results in dilated lymphatic sacs and subsequent lymphangioma formation. Other possible hypotheses propose acquired factors such as trauma, fibrosis, and inflammatory etiologies [10].

Abdominal cystic lymphangioma (ACL) may occur anywhere along the course of the gastrointestinal tract and visceral organs. ACL has been found to occur in the small bowel mesentery in 80% of cases, while retroperitoneal location appears to be less common [11]. Abdominal distension, abdominal pain, and other symptoms of intestinal obstruction may accompany ACL, but the incidence of proven intestinal obstruction is actually quite low. Abdominal pain (72.3%) and distension (34%) are the most commonly experienced symptoms with ACL [12, 13]. Abdominal pain was noted in two of the cases, while abdominal distension was observed in all three cases of intra-abdominal lymphangioma. ACL may be asymptomatic and may be found incidentally during imaging for another pathology [8].

Axillary lymphangiomas typically present as painless swellings, which are soft, compressible, nontender, and transilluminant and are without a bruit on physical examination [14]. Axillary swellings may pose a diagnostic challenge initially with some patients being misdiagnosed as lipoma, neurofibroma, haematoma, gynaecomastia, and dermoid cyst [15]. The patient in case 4 was initially thought to have unilateral gynaecomastia at birth, but USS soon after made the diagnosis evident. Although children may present with axillary swellings at different ages, it is believed that the lymphatic malformation is present from birth. It is especially difficult to get complete excision of axillary lymphangiomas without disrupting developing breast tissue in females. In such cases, parents should be extensively counseled on cosmetic outcome.

Axillary lymphangiomas can be complicated by infection usually secondary to a respiratory tract infection. Infected lesions become red and tender and the patient may become pyretic. In some cases, they may turn into abscesses and require incision and drainage [14]. Another common complication of axillary lymphangioma is spontaneous bleeding into the cyst [14] as seen in case 4. ACL may also be complicated by infection or haemorrhage within the cyst [13] also seen in case 1. We believe that haemorrhage within cysts may have been responsible for patient's anaemia in case 1.

Radiological investigations greatly aid in the preoperative diagnosis of lymphangioma with ultrasound scan (USS) being used as the initial mode of investigation, detecting the lesion in 100% of cases in one study [16]. In cases of axillary lymphangioma, USS helps in differentiating glandular from cystic tissue [15]. Macrocystic lymphangiomas typically appear as anechoic cavities with septae and debris on gray scale USS [7]. Ultrasound scan is primarily used to monitor size and determine extent preceding excision [7]. However, computed tomography (CT) scan is significantly better at determining tumour content [13]. CT is especially helpful in determining the relations to major vessels and other surrounding structures and hence is essential in planning the surgery [13]. Lymphangiomas appear as low attenuation and fluid-filled masses on CT. Fluid-fluid levels can occasionally be seen representing acute or subacute bleeding into the cyst [7]. Magnetic resonance imaging (MRI) is an excellent modality to assess lesion extent in terms of tissue planes, airway compression, mediastinal extension, and potential solid organ and bone involvement [7]. Abdominal X-ray is noncontributory in diagnosing ACL but can detect intestinal obstruction associated with ACL [11]. The combination of both USS and CT seems to provide as much information as that obtained from MRI in ACL.

Surgical excision is known to be the gold standard for treatment of all types of lymphangioma and hence was the mode of treatment in all of the above cases. However, incomplete surgical resection is linked with recurrence as seen in case 1. Recurrence rate accounts for 10% of cases due to difficulty in resecting the entire cyst wall and is especially so for retroperitoneal cysts due to close relationship to vital retroperitoneal structures which make resection very hazardous or even impossible [11]. Postoperative complications in cases of ACL include peritonitis, haemorrhage, abscess, and torsion, which is a rare postoperative complication [10]. Wound infection, haemorrhage, hypertrophied scar, and lymphatic discharge from the wound are postoperative complications of axillary lymphangioma excision [14].

Use of sclerosing agent such as bleomycin at the time of operations remains controversial but may have some benefit in management of extra-abdominal lymphangioma [11]. Ozeki et al. recently reported some success in the use of propranolol in the treatment lymphatic malformation. Cases of diffuse intractable lymphangiomatosis showed very promising results with the use of propranolol, which is thought to cause downregulation of the Raf mitogen activated protein kinase signaling pathway, with reduced expression of vascular endothelial growth factor (VEGF) [17]. This led to a decrease in size of up to 30.6% in patients treated with propranolol [18].

Laparoscopic resection of ACL is not well documented in children but Son and Liem strongly advocate that laparoscopic surgery is both safe and feasible in resection of ACL in children. In their study of 47 children with ACL, there were no reports of mortality or intestinal obstruction postoperatively and there was a conversion rate of only 6.8%. They suggest that many cases can in fact be performed by a single port technique [13].

The diagnosis of lymphangioma is confirmed by histology, which shows benign cystic proliferation of lymphatic tissue. However typically they do not communicate with the lymphatic system (Figure 4). Immunohistochemistry may be used to differentiate intra-abdominal hemangioma from lymphangioma in cases of diagnostic uncertainty. Lymphatic malformations are thin walled vessels lined by lymphatic endothelial cells that are immunohistochemically positive for endothelial markers D2-40 and lymphatic vessel endothelial receptor 1 [9].

There is very limited regional data regarding lymphangioma. Only two regional publications exist,* Intrathoracic cystic hygroma in an infant with respiratory failure*, Balkaran et al. (Trinidad) [19], and* Orbital lymphangioma in a child: a diagnostic dilemma*, Mowatt and Crossman (Jamaica) [20]. No published regional data regarding axillary and intra-abdominal cystic lymphangioma could be found in our literature search. We do hope that this paper may serve to increase understanding of presentation and treatment of lymphangioma in the Caribbean region.

## 4. Conclusion

This paper discusses a range of extracervical lymphangioma cases seen at San Fernando General Hospital, Trinidad. We report three intra-abdominal cases, and the most common clinical presentations were abdominal pain and distension. Also two axillary cases were reported, which presented as painless axillary masses. The major concerns for excision of axillary lymphangioma by parents and surgeons were cosmesis and feasibility of complete resection without disruption of developing breast tissue and axillary vessels. We believe that ultrasound scan is very good at detection of the lesion, while CT is better at determining tumour content and planning for the operation. It is our opinion that complete surgical excision can be achieved.

## Figures and Tables

**Table 1:** Comparison of cases.

Case	Age	Gender	Site of lymphangioma	Signs/symptoms on presentation	Treatment	Response to treatment
Case 1	6 yrs	F	Retroperitoneal	Periumbilical pain, vomiting, abdominal distension, and constipation	Surgical excision	Recurrence following resection at 6 weeks of life resulting in second surgery at 6 years. Yearly follow-up with USS for the past 9 years; no recurrence

Case 2	4 yrs	M	Retroperitoneal	Periumbilical pain, constipation, abdominal distension, and left flank firmness	Complete surgical excision	Yearly follow-up with USS for the past 8 years; no recurrence

Case 3	4 yrs	F	Mesenteric	Progressive abdominal distension	Complete surgical excision	Yearly follow-up with USS for the past 3 years; no recurrence

Case 4	8 yrs	F	Left axillary	Axillary swelling	Complete surgical excision with conservation of developing breast tissue	Yearly follow-up with USS for the past 3 years. No recurrence and normal symmetrical breast development

Case 5	14 months	M	Right axillary	Swelling on the anterior chest wall since birth with increasing size	Observation for the first year followed by complete surgical excision due to progressive increase in size	Yearly follow-up with USS for the past 2 years; no recurrence

M: male and F: female.
